# A systematic review of the measurement properties of patient reported outcome measures used for adults with an ankle fracture

**DOI:** 10.1186/s41687-019-0159-5

**Published:** 2019-12-17

**Authors:** Rebecca McKeown, David R. Ellard, Abdul-Rasheed Rabiu, Eleni Karasouli, Rebecca S. Kearney

**Affiliations:** 1 Warwick Clinical Trials Unit, Warwick Medical School, Clinical Sciences Research Laboratories, University of Warwick, University Hospitals Coventry and Warwickshire, Coventry, CV2 2DX UK; 2 Trauma and Orthopaedics Department, King’s College Hospital, Denmark Hill, London, SE5 9RS UK

**Keywords:** Ankle fracture, Patient reported outcome measures, Measurement properties, Systematic review, Validity, Reliability

## Abstract

**Background:**

Ankle fractures are painful and debilitating injuries that pose a significant burden to society and healthcare systems. Patient reported outcome measures (PROMs) are commonly used outcome measures in clinical trials of interventions for ankle fracture but there is little evidence on their validity and reliability. This systematic review aims to identify and appraise evidence for the measurement properties of ankle specific PROMs used in adults with an ankle fracture using Consensus Based Standards for the Selection of Health Measurement Instrument (COSMIN) methodology.

**Methods:**

We searched MEDLINE, Embase and CINAHL online databases for evidence of measurement properties of ankle specific PROMs. Articles were included if they assessed or described the development of the PROM in adults with ankle fracture. Articles were ineligible if they used the PROM to assess the measurement properties of another instrument. Abstracts without full articles and conference proceedings were ineligible, as were articles that adapted the PROM under evaluation without any formal justification of the changes as part of a cross-cultural validation or translation process. Two reviewers completed the screening. To assess methodological quality we used COSMIN risk of bias checklist and summarised evidence using COSMIN quality criteria and a modified Grading of Recommendations Assessment, Development and Evaluation (GRADE) approach. Two reviewers assessed the methodological quality and extracted the data for a sample of articles.

**Results:**

The searches returned a total of 377 articles. From these, six articles were included after application of eligibility criteria. These articles evaluated three PROMs: A-FORM, OMAS and AAOS. The A-FORM had evidence of a robust development process within the patient population, however lacks post-formulation testing. The OMAS showed sufficient levels of reliability, internal consistency and construct validity. The AAOS showed low quality evidence of sufficient construct validity.

**Conclusions:**

There is insufficient evidence to support the recommendation of a particular PROM for use in adult ankle fracture research based on COSMIN methodology. Further validation of these outcome measures is required in order to ensure PROMs used in this area are sufficiently valid and reliable to assess treatment effects. This would enable high quality, evidenced-based management of adults with ankle fracture.

## Background

Ankle fractures cause significant pain, reduced mobility and subsequent limitation of usual activities [1]. The injury overall demonstrates a bimodal distribution, most commonly affecting young active males and older females. However some fracture patterns, such as more severe bi-malleolar and tri-malleolar ankle fractures demonstrate a unimodal distribution, most commonly affecting an older female population, indicative of being an osteoporotic injury [2, 3]. Epidemiological studies have shown that the incidence of ankle fractures is rising, likely due to the ageing population, many of whom continue to remain physically active into later life [4, 5]. Ankle fractures contribute to the increasing health and social care costs that accompanies an ageing population, specifically the cost of managing fragility fractures [6]. This cost was approximately €37.5billion across six European countries in 2017; a figure that is forecasted to rise to €47.4 billion by the year 2030 [7]. Fractures of the lower limb have a significant impact on the lives of individuals affected, not only on mobility and usual activities but they have also been linked to the development of anxiety and depression [8]. Evidence based treatment of burdensome and prevalent injuries such as ankle fractures is important, yet there is a lack of consensus surrounding the optimal management strategies for this injury [9]. It is therefore of paramount importance that funding bodies continue to allocate resources for the conduct high quality clinical trials in order to establish the most cost-effective management strategies for ankle fractures [9, 10].

Clinical trials of interventions for fractures of the lower limb often utilise Patient Reported Outcome Measures (PROMs) as primary outcomes [11–13]. It is important that the instruments used to measure treatment effects in clinical trials demonstrate adequate measurement properties, such as validity, reliability and responsiveness, for the population they intend to assess. However, there is evidence that some widely used PROMs in trauma and orthopaedic research lack evidence for their measurement properties [14].

Conducting a randomised controlled trial is expensive, time consuming and relies on the good will of participants to be randomised to an intervention and complete questionnaires. If the PROM used in a clinical trial does not measure the treatment effects of the interventions in a valid and reliable way, this places the unnecessary burden of randomisation and trial processes onto participants. Using PROMs with insufficient measurement properties in randomised controlled trial is therefore a waste of resource and unethical [15]. A systematic review assessing the psychometric properties of PROMs for ankle fracture has been completed previously [16], which concluded that the Ankle Fracture Outcome of Rehabilitation Measure (A-FORM) was the most appropriate measure to use. However, considering the small number of articles included in this review, the growing incidence of ankle fractures and subsequent need for research in this area, an update is deemed timely, with a particular focus on PROMs currently and previously used in randomised controlled trials of interventions for ankle fractures.

The aim of this review is to identify and critically appraise the available evidence for the measurement properties of foot and ankle specific PROMs for use in adults with an ankle fracture. The results of this review will aim to determine the most appropriate instrument for use in evaluating change resulting from interventions in the context of randomised controlled trials in this research area.

## Methods

We prospectively registered this review with PROSPERO International Prospective Register of Systematic Reviews (Reference CRD42018103112). Consensus Based Standards for the Selection of Health Measurement Instrument (COSMIN) Methodology for Systematic Reviews of Measurement properties of PROMs was adhered to [15] and this review utilises definitions according to published COSMIN consensus based terminology [17]. This systematic review is reported using the Preferred Reporting Items for Systematic Reviews and Meta-Analyses (PRISMA) checklist (Additional file 2)[18].

This review was completed following a previous systematic review looking to assess all outcome measures collected in clinical trials of interventions for ankle fracture [19]. The outcome measures included all both primary and secondary outcome measures and we formulated a comprehensive list of all ankle specific PROMs collected. These PROMs formed the pre-specified list we used to identify evidence for and evaluate during this current review. The PROMs on the pre-specified list being evaluated in this review are: the AAOS Foot and Ankle Outcome Questionnaire (AAOS) [20], the Ankle Fracture Outcome of Rehabilitation Measure (A-FORM) [21], the Foot and Ankle Ability Measure (FAAM) [22], the Karlsson Score (KS) [23], the KOOS Foot and Ankle Outcome Survey (FAOS) [24] the Manchester-Oxford Foot and Ankle Questionnaire (MOXFQ) [25] and the Olerud Molander Ankle Score (OMAS) [26].

### Eligibility criteria

Included articles assessed the measurement properties, development or interpretability of one or more of the PROMs included in the pre-specified list in a majority patient population of adults with ankle fracture. Here, majority is defined as equal to or greater than 50% of the sample. In articles which did not reach the criteria of 50% but performed a separate analysis on the ankle fracture sub-sample of patients, these articles were included and only the analyses performed on the single sub-sample of individuals with ankle fracture were included; any analyses on the sample as a whole or comparing the two clinical groups were not included.

Articles were ineligible for inclusion if they use the PROM/s only for outcome measurement in an experimental study, where no formal evaluation of a measurement property is completed. Articles which use the PROM in question to validate another PROM (not on the pre-specified list here) were also ineligible for inclusion. Studies were excluded if the authors adapted the PROM in any way without formal justification of the changes as part of a translation or cross-cultural validation process. Abstracts without full articles and conference proceedings were not eligible for inclusion.

### Search strategy and study selection

A systematic search of the literature was completed using the MEDLINE, EMBASE and CINAHL databases on 16/04/2019 up to the present date with no date limits applied using search strategies developed by the COSMIN group specifically for this type of review [27]. Additional file 1 details the search strategies. We also reviewed the reference lists of all included studies for any other potentially eligible papers for inclusion.

The lead author and a second reviewer (AR) independently screened the articles by title and abstract for possible inclusion. The reviewers selected any articles which were potentially eligible from title and abstract review and retrieved the full text. If it was unclear at the initial title and abstract review, the full text was retrieved and reviewed for purposes of completeness. If at least one of the reviewers felt that a study might be eligible based upon the initial title and abstract screening, then both researchers independently reviewed the full text to assess eligibility for inclusion. The reviewers then discussed findings and reached consensus on inclusion of articles. In instances of disagreement, a third reviewer (RSK) was consulted for a final decision.

### Assessment of methodological quality and assessment of measurement properties

The methodological quality of the articles included in this review was assessed using the COSMIN risk of bias checklist [28]. Evidence for the measurement properties in the included articles was extracted and assessed against the COSMIN criteria of good measurement properties. The overall evidence from all articles was pooled and summarised using the modified Grading of Recommendations Assessment, Development and Evaluation (GRADE) quality of evidence method [15]. The assessment of methodological quality and the data extraction was completed for all articles by the lead author initially. A second reviewer (EK) independently reviewed the methodological quality and performed data extraction in a sample of the articles (> 50%) to ensure a reduction of bias in the methodological quality assessment and data extraction process. Following independent review, authors discussed their results and reached consensus. When unable to reach a consensus, a third reviewer (RSK) was consulted for a final decision.

A decision was made that the criteria and box for criterion validity was not to be completed as there is no accepted gold-standard measure for assessing outcome in adults with ankle fracture, therefore this measurement property does not apply in this particular case. If reported, data on the interpretability and feasibility of the PROMs were also extracted and reviewed. We contacted developers of the PROMs where possible to obtain a copy of the user manual (if available) and to ensure that, to their knowledge, there were no further validation studies on the scores which may not have been identified in the database searches.

### Hypotheses for construct validity

Hypotheses for assessing the construct validity evidence in the instances that this was assessed in the included articles was pre-defined [29]. The following thresholds of correlation were used for the hypothesis setting:

1. A weak correlation is defined as < 0.30
2. A weak to moderate correlation is defined as > 0.20 - < 0.40
3. A moderate correlation is defined as > 0.30 - < 0.70
4. A moderate to high correlation is defined as > 0.60 to < 0.80
5. A high correlation is defined as > 0.70

The hypotheses tested during this review for construct validity are outlined in Table 1:

**Table 1:** Hypotheses set for construct validity testing

Hypothesis Number	Hypothesis
1	Correlation with scores of instruments measuring a similar construct or another PROM included in the pre-specified list will be highly or moderately to highly correlated.
2	Correlation with scores of instruments measuring related but not the same constructs, for example generic disability scores or health related quality of life measures will be either moderately to highly or moderately correlated.
3	A weak to moderate correlation will be observed between PROM/s scores of instruments included here and two different subgroups of patients. These subgroups will be individuals who have had their fracture managed operatively and those who have had their fracture managed non-operatively. Here, fracture management is used as a surrogate for severity of fracture (i.e. more severe fractures usually managed operatively). Therefore, we would expect to see a weak to moderate correlation between the PROM score and the severity of the fracture.

## Results

### Search results

**Fig. 1:**
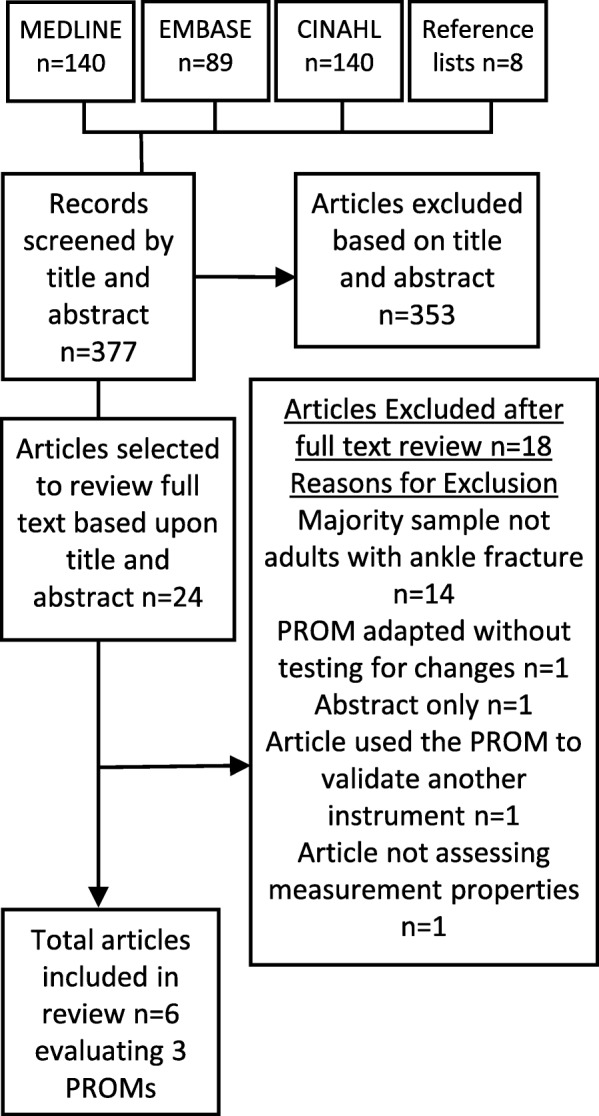
PRISMA flow diagram

The searches produced a total of 377 returns. Following initial screening of the titles and abstracts, 353 records were excluded, leaving 24 articles for full text review. Following full-text review of the 24 articles, six articles were included in this review [30–34] and details of the application of the eligibility criteria can be found in the PRISMA Diagram in Fig. 1. The included six articles assessed three of the eight pre-specified PROMs; the AAOS, A-FORM and OMAS. There was no evidence for the measurement properties of the remaining PROMs in the pre-specified list (FAAM, FAOS, KS and MOXFQ) in the population of adults with ankle fracture.

### Characteristics of included PROMs

Table 2 shows the characteristics of the PROMs included in this review.

**Table 2:** PROM characteristics

PROM	Construct(s)	Target Population	Recall period	Items and subscales	Response options	Source language (and additional language versions)
AAOS	Outcome for foot or ankle disability	Individuals with foot or ankle disability	Past 1 week (or since injury if less than 1 week)	25 items, 20 items in the core scale and a shoe comfort scale consisting of 5 items	Likert scales varying in length 1–3, 1–5, 1–6 or 1–7	English(Mexican- Spanish)
A-FORM	Outcome following ankle fracture	Individuals with ankle fracture	No recall period given	15 items, no sub-scales. Two parts to the questionnaire.	Single response, multiple choice - 5 response options	English
OMAS	Outcome following ankle fracture	Individuals with ankle fracture	No recall period provided	9 items, no subscales	Single response, multiple choice - 2, 3, 4 or 5 response options	Not specified (English, Turkish and Norwegian)

All of the PROMs included in this review are paper based questionnaires self-administered by the patient either in a clinical or research context. The AAOS consists of 25 questions including stiffness (one item), swelling (one item), pain (nine items), giving way (three items), function (six items) and footwear (five items). The score consists of a core score (AAOS-CS) comprising of 20 items and a shoe comfort scale (AAOS-SCS) comprising of five items. The scores are calculated to a normative score for each of these two scales, which is then converted to a summative mean for both the AAOS-CS and AAOS-SCS. The summative score for each subscale ranges between 0 and 100 with higher scores indicating a more favourable outcome.

The A-FORM consists of 15 items including pain, swelling, stiffness, anxiety regarding footwear, sleeping, jumping, waking, social aspects, anxiety related to future ankle function, depression and fatigue. The raw score is converted to a summary score which ranges between 0 and 100, with lower scores indicating more favourable outcomes. The footwear item is not included in the summary score conversion, so users are asked to omit this item from the summary score conversion process. The summary score conversion table is found in the user manual which can be requested from the developers at no cost to users. The summary score conversion was based on the Rasch analysis presented in the development article included in this review [32].

The OMAS is a nine-item questionnaire including pain, stiffness, swelling, stairs, squatting, supports, jumping, running and usual activities. Final scores range between 0 and 100 with higher scores indicating more favourable outcomes. The score is totalled using the scoring system provided in the development paper included in this review [26]. Different items of the score provide varying numbers of points which contribute to the overall score. For example, the item for pain is awarded between 0 and 25 points depending on the answer selected, work and activities of daily living between 0 and 20 points and squatting between 0 and 5 points.

### Study characteristics and methodological quality assessment

Table 3 shows the characteristics of the six studies included in this review. As Table 4 demonstrates, none of the articles included here scored higher than adequate on the methodological quality assessment checklist. Whilst several articles [30–34] translated the PROM and then performed analyses of measurement properties on the translated PROM, these studies did not cross-culturally validate the translated PROMs using an analysis of measurement invariance. Therefore, it was not possible to determine any differences in scores secondary to cultural contextual factors and the box for cross-cultural validity was not deemed to be relevant in these instances. The developers of the A-FORM instrument [21] did perform an assessment of internal consistency using Cronbach’s alpha and structural validity using a Rasch Analysis, however these analyses were not completed on the final set of questions but on a larger set of the initial items for purposes of determining inclusion in the questionnaire. Therefore, this article was not scored for internal consistency and structural validity in this case as these analyses were completed for purposes of item reduction.

**Table 3:** Characteristics of studies

	Population					
Article, PROM and Language	Number of participants (n)	Age mean (±, range) (yrs)	Gender (% female)	Injury Information	Follow up duration mean (±, range)	Method of Collection
Buker et al. (2018) OMAS, Turkish	91	41.54 (±13.28, 20–60)	30.8%	Operatively managed ankle #s	27.92 months (±8.94, range N/S)	Initial in clinic, follow up in clinic or on telephone
Garratt et al. (2018) OMAS, Norwegian	Cohort 959, 299 for test-retest questionnaire	57.5 (± N/S, 22.2–91.2)	56.8%	Operatively managed ankle #s	Not specified, stated recruited over a 3 year period	At home via post
McPhail et al. (2014) A-FORM, English	Delphi panel – 8 Cohort - 41	36.8 (± N/S, 26.1–53.8)	27%	Operatively managed (46.3%) and non-operatively managed (53.7%) ankle #s	6–8 week post injury and at 12–16 weeks post injury	Either in clinic or at home via post
Olerud and Molander (1984) OMAS, Language N/S	90	N/S	N/S	Operatively managed ankle #s	N/S	N/S
Turhan et al. (2018) OMAS, Turkish	100	42.3 (±17.7, 16–81)	49%	Operatively (57%) and non-operatively managed (43%) ankle #s	4.3 years (± and range N/S)	N/S
Zelle et al. (2017) AAOS, Spanish	100 (83 returned 1st questionnaire, 63 returned 2nd questionnaire)	42.98 (± N/S,18–88)	41%	58 ankle #s, 5 talus #s, 1 Achilles tendon rupture, 11 calcaneus #s, 6 midfoot #s. 73 operatively managed and 27 non-operatively managed	3.97 months (±4.71 range N/S)	Initial at clinic or via post, follow up was via post.

Key: N/S = not specified, ± = standard deviation, # = fracture; shows the characteristics of the six studies included in this review. Table 4 shows the overall methodological quality for each measurement property assessed in each of the articles using the COSMIN Risk of Bias Checklist [28]. The four articles which underwent the second review process for both risk of bias assessment and data extraction, following COSMIN guidance, are marked on the table with an asterisk

**Table 4:** Scores for methodological quality using COSMIN risk of bias checklist

PROM	AAOS	A-FORM	OMAS			
Article	*Zelle* et al. (2017) *	*McPhail* et al. (2014) *	*Buker* et al. (2017) *	*Garratt* et al. *(2018)*	*Turhan* et al. (2017) *	*Olerud and Molander (1984)*
PROM Development		Doubtful				Inadequate
Content Validity						
Structural Validity				Doubtful		
Internal Consistency			Doubtful	Doubtful	Doubtful	
Cross cultural validity and measurement invariance						
Reliability	Inadequate		Inadequate	Doubtful	Inadequate	
Measurement Error				Doubtful	Doubtful	
Criterion Validity	N/A	N/A	N/A	N/A	N/A	N/A
Construct validity	Doubtful (Convergent validity)		Doubtful (Convergent validity)	Adequate (Convergent Validity) Doubtful (Known Groups Validity)	Adequate (Convergent validity)	
Responsiveness						

Scores for methodological quality using COSMIN Risk of Bias Checklist; available options are very good, adequate, doubtful, inadequate or N/A. Key: * = Articles were assessed by second reviewer for risk of bias and data extraction, N/A: Not applicable. A blank box indicates that the measurement property was not assessed in the study

Following the COSMIN guidance for PROM development, an article encountered in the reference list of the A-FORM development articles [32] was taken into consideration as it involved the development of the A-FORM [1]. Whilst this article did not meet the inclusion criteria of the review, the review team felt this article provided important developmental work for the PROM, therefore the information presented in this article was included when completing the box for PROM development of the A-FORM.

### Measurement properties

Table 5 shows the results presented for each of the measurement properties in the included articles in this review. Table 6 shows the summary of findings table, demonstrating the overall evidence for measurement properties against the COSMIN GRADE Assessment.

**Table 5:** Results presented in articles

Article and PROM	Structural Validity	Internal Consistency	Cross-cultural validity	Reliability	Measurement Error	Construct Validity	Responsiveness
Zelle et al. (2017) AAOS	N/R	N/R	N/R	ICC or weighted kappa not reported	N/R	AAOS-CS with SF-36-PCS *r* = 0.667 AAOS-CS with SF-36-MCS *r* = 0.506 AAOS-SCS with SF-36-PCS *rs* = 0.358 AAOS SCS with SF-36-MCS *rs* = 0.356	N/R
McPhail et al. (2014) A-FORM	N/R	N/R	N/R	N/R	N/R	N/R	N/R
Buker et al. (2017) OMAS	N/R	Cronbach’s Alpha 0.76	N/R	ICC 0.98	N/R	OMAS with 5 FAOS Subscales: pain *r* = 0.788, symptoms *r* = 0.753, ADL *r* = 0.798, sports *r* = 0.809, QoL *r* = 0.772	N/R
Garratt et al. (2018) OMAS	CFI 0.99 and TLI 0.98	Cronbach’s Alpha 0.82	N/R	ICC 0.92	MIC not defined	OMAS with SEFAS *rs* = 0.88 OMAS with SF-36-PCS *rs* = 0.77 OMAS with EQ-5D *rs* = 0.79	N/R
Olerud and Molander (1984) OMAS	N/R	N/R	N/R	N/R	N/R	N/R	N/R
Turhan et al. (2017) OMAS	N/R	Cronbach’s Alpha 0.84	N/R	ICC 0.98	MIC not defined	OMAS with FAAM-ADL *r* = 0.86 OMAS with FAAM-S *r* = 0.83 OMAS with SF-12-PCS *r* = 0.72 OMAS with SF-12-MCS *r* = 0.60	N/R

*Key: r = Pearson’s correlation, rs = Spearman’s correlation, ADL = Activities of Daily Living, QoL = Quality of life, FAAM-ADL = FAAM Activities of Daily Living Subscale, FAAM-S – FAAM Sports Subscale, AAO-CS = AAOS Core Score, AAOS-SCS = AAOS Shoe Comfort Scale, PCS=Physical component Score, MCS = Mental component Score, EQ-5D = EuroQol EQ-5D-5 L Score, ICC=Intraclass correlation coefficient, TLI = Tucker Lewis index, CFI=Confirmatory Factor Analysis, MIC = Minimally Important Change*

**Table 6:** Summary of findings table

PROM	AAOS		A-FORM		OMAS	
	Overall Rating	Quality of Evidence	Overall Rating	Quality of Evidence	Overall Rating	Quality of Evidence
Content validity	?	N/A	?	N/A	?	N/A
*Relevance*	?	N/A	?	N/A	?	N/A
*Comprehensiveness*	?	N/A	?	N/A	?	N/A
*Comprehensibility*	?	N/A	?	N/A	?	N/A
Structural validity	?	N/A	?	N/A	+	High
Internal consistency	?	N/A	?	N/A	3+	Moderate
Cross-cultural validity	?	N/A	?	N/A	?	N/A
Measurement invariance	?	N/A	?	N/A	?	N/A
Reliability	?	Very Low	?	N/A	3+	Low
Measurement Error	?	N/A	?	N/A	?	N/A
Criterion validity	N/A	N/A	N/A	N/A	N/A	N/A
Construct validity	4+	Low	?	N/A	16+	High
Responsiveness	?	N/A	?	N/A	?	N/A

*Key: + = Sufficient,? = Indeterminate, − = Insufficient, N/A = not applicable*

The AAOS demonstrated low levels of evidence for sufficient construct validity. Zelle et al. [34] correlated the scores of the AAOS-CS and AAOS-SCS with the scores of the SF-36 subscales: the Physical Component Score (PCS) and Mental Component Score (MCS). The results of these four correlations performed met hypothesis 2 of the pre-defined hypotheses detailed in Table 1. The authors also assessed the test-retest reliability of the translated questionnaire, however, this result was indeterminate for this measurement property as the ICC or weighted Kappa were not reported in the results.

McPhail et al. [21] detailed the development of the A-FORM through completion of item reduction exercises including a Delphi study and Rasch analysis. The development of the article was thorough and included both patients and clinicians in the concept elicitation interviews and the item-reduction Delphi exercise. However there was a gap in the evidence here with regards to content validity as there was no cognitive interview testing done on the final version of the questionnaire to assess relevance and comprehensiveness of the instrument, therefore the content validity box was not completed [35].

Authors of the included studies assessed the translated versions of the OMAS for structural validity in Norwegian and internal consistency, reliability and construct validity in both Norwegian and Turkish languages. The OMAS Norwegian version achieved high level evidence for sufficient construct validity; Garratt et al. [33] correlated the OMAS scores with the scores of the Self-Reported Foot and Ankle Score (SEFAS) which met hypothesis 1 of the pre-defined hypotheses in Table 1. They also correlated the OMAS scores with the EQ-5D and the SF-36 scores respectively, both of which met hypothesis 2 of those pre-defined in Table 1. The Norwegian OMAS achieved high level evidence for sufficient structural validity. The OMAS in both Buker et al. [30] and Turhan et al. [31] correlated the scores of the Turkish version of the OMAS with various patient reported outcome measures, all of which met hypotheses 1 or 2 in the predefined hypotheses in Table 1. Turkish and Norwegian versions achieved low-level evidence for sufficient reliability where reported. Both The OMAS was assessed for the measurement error through assessment of the minimal detectable change however as no data is available on the minimal important change for this PROM, results for this measurement property were indeterminate against COSMIN criteria.

### Interpretability and feasibility

Table 7 shows the information reported in the articles on the interpretability and feasibility of the PROMs included in this review.

**Table 7:** Interpretability evidence of the PROMs

Article and PROM	Distribution of total scores in study population	Percentage of missing total scores	Percentage of missing items	Floor and Ceiling Effects (Interpretability)
Zelle et al. (2017)* AAOS	Normal distribution following Shapiro-Wilks Test -no Mean (±) provided.	Missing total scores 83 of 100 in first test and 63 of 100 in re-test.	No data reported on items missing.	Not reported
McPhail et al. (2014)* A-FORM	Not reported for questionnaire in final format	Not reported	Not reported for questionnaire in final format	Not reported
Olerud and Molander (1984) OMAS	Not reported	Not reported	Not reported	Not reported
Garratt et al. (2018) OMAS	75.62 (±24.07) - No information on distribution	1.6% missing	17.3% of respondents missed at least one item. “Jumping” most commonly missed item (6.2%).	Not reported
Buker et al. (2017)* OMAS	72.58 (±23.27) - No information on distribution	Not reported	Not reported	Not reported
Turhan et al. (2017)* OMAS	74.1 (±23.7) - No information on distribution	Not reported	Not reported	Floor - 0% Ceiling - 27-29%

Key: * = * = Articles were assessed by second reviewer for risk of bias and data extraction

There was no information reported in any of the included studies on response shift or minimal importance difference of the measures therefore these facets of interpretability have not been included in Table 7. Some articles did not report any data on the interpretability of the scores evaluated. Whilst the majority of articles included here do not report aspects of feasibility in there research, throughout the process of the review, we could conclude that they were all available free of charge without the need to purchase a licence. The instruments are easy and relatively quick to complete in a clinic setting or remotely and returned in the post, placing minimal burden on participants completing them. We found no information or guidance available on any of the included PROMs regarding completion electronically or via telephone. Like most questionnaires, the PROMs included here require the ability to read, comprehend and respond to the questions, with no evidence found during this review of these instruments being suitable for measurements by proxy.

COSMIN methodology advises that in order to recommend a PROM, it should demonstrate any level of content validity and a minimum of low level evidence for internal consistency [15]. None of the instruments included in the review have met this criteria, therefore we are unable to recommend any of these PROMs for use in this patient population. However, there is no evidence of insufficient measurement properties in these PROMs, therefore further validation studies are required before they can be recommended for use in this patient population [15].

## Discussion

This review demonstrates that at the time this review was undertaken, none of the PROMs used in clinical trials of interventions for ankle fracture had adequate evidence of measurement properties and we are therefore unable to recommend a particular PROM for use in this context and patient population. Furthermore, there were four additional PROMs (FAAM, FAOS, KS, and MOXFQ) which have been or are currently being used in clinical trials of interventions for ankle fracture for which the current review did not find any evidence of their measurement properties within the patient population. Whilst the OMAS demonstrates sufficient internal consistency, structural validity and construct validity, the PROM development scored poorly against COSMIN criteria used in this review. In contrast, the A-FORM demonstrates some evidence for PROM development within the patient population, but there is limited post-formulation testing of this PROM.

This review updates the one completed in 2016 by Ng et al. [16] which assessed the psychometric properties of PROMs for ankle fractures. The current review includes four additional recently published articles and focussed on only ankle specific PROMs, whereas the previous review also included articles assessing both ankle and generic health-related quality of life PROMs. This review differs in that we used a pre-specified list of ankle specific PROMs which have been and are currently used in clinical trials for ankle fracture interventions. Ng et al. [16] recommended the use of the A-FORM suggesting it has a robust development process within the patient population. Whilst we agree that the A-FORM has more a more adequate development process when compared to other PROMs presented here, we do not think it is appropriate for recommendation due to the lack of evidence of sufficient internal consistency of the final version of the instrument. This is based on the updated COSMIN guidance on systematic reviews of this nature. Other studies have completed similar reviews on outcome measures used in generic foot and ankle research with similar results presented. A review assessing all foot and ankle PROMs for use in any foot and ankle disorder concluded that there was no region specific outcome measure with appropriate levels of evidence for their measurement properties for use in individuals with foot and ankle disorders [36].

Strengths of this review include the use of a well-developed, thorough and consensus based methodology and search filters for finding and reviewing the evidence for development and measurement properties of PROMs. Limitations of the review include the inherent difficulty in defining the construct under analysis; there is little research into the experiences of individuals recovering from an ankle fracture and further research into the construct of interest would be beneficial. The construct of outcome in ankle fracture recovery may vary depending on several individual factors, such as age, gender and whether the fracture is treated operatively or non-operatively. When considering the varied distributions of the different ankle fracture patterns which has been demonstrated in the epidemiological literature [3], one could argue that osteoporotic fractures in older adults are a different injury to those sustained by younger adults. Subsequently, the construct in question between these two different patient groups might vary considerably and may require different PROMs or versions of PROMs. Furthermore, the articles included here assessed differing populations with regard to fracture management; some assessed only operatively managed ankle fractures [26, 30, 33] and others included a mixture of operatively and non-operatively managed fractures [21, 31]. One article also included non-ankle fractures patients, which may have further confounded the results for the measurement properties assessed here [34]. Four of the included articles here were concerned with the OMAS [26, 30–33], only one article did so for the AAOS [34] and another one for the A-FORM [21], making it difficult to compare evidence between the three PROMs.

We encountered difficulty in applying the COSMIN methodology and assessment criteria to older articles such as the development of the OMAS instrument [26]. We acknowledge that the age of an instrument does not excuse it from critical review and analysis and further research into the acceptability of these instruments to patients is warranted to inform the ongoing use of older PROMs.

## Conclusions and implications

This review shows that currently there is no PROM that can be recommended for use for the purpose of assessing outcome in clinical trials of interventions for ankle fracture. Further validation work should focus on ascertaining the acceptability, relevance and comprehensiveness of commonly used questionnaires such as OMAS in a population of adults with ankle fracture. Future research studies in this area should make use of COSMIN based standards for designing and reporting validation research to ensure that the appropriate evidence base is acquired for a PROM to be recommended. As this review demonstrates, there is no evidence that this PROM was formulated with the input of individuals who have ankle fractures and understanding the content validity of this widely used instrument would enable an understanding of whether it is fit for purpose in the patient population or whether the use of this outcome measure should be discontinued. Furthermore, the OMAS demonstrated ceiling effects in excess of the widely recognised acceptable level of 15% [37, 38], which warrants further investigation.

Future exploratory research should aim to understand the patient experience of ankle fracture and the factors of most importance to individuals with this injury, with an understanding that this may differ between age group of the individuals and possibly fracture management. It might well be that the construct between these groups differs so much that it is not appropriate for the same PROM to be used between these populations. Exploring the relevance and comprehensiveness of PROMs such as the OMAS which were not developed with input from the patient population would be beneficial to ascertain the appropriateness of the ongoing use of these outcome measure. None of the articles here assessed the responsiveness of the PROMs and future research should seek to ensure that the instruments are suitably responsive to detect treatment effects in resource-intensive clinical trials. Furthermore, validation of the A-FORM questionnaire to ascertain the measurement properties of this PROM in its final format would be advantageous. Further validation research of the PROMs used in ankle fracture is warranted here to ensure that randomised controlled trials in this clinical area answer the questions needed to manage these individuals most effectively. Furthermore, the preparation of an agreed core outcome set for use in this patient population would be advantageous, enabling the conduct of high quality trials using an appropriate and standardised set of outcome measures for this important injury.

## Additional file

**Additional file 1.** Search strategies.

**Additional file 2.** PRISMA Checklist.

## Data Availability

All data generated or analysed during this study are included in this published article and its supplementary information files.

## Abbreviations

AAOS: American Academy of Orthopaedic Surgeons Foot and Ankle Outcome Questionnaire
AAOS-CS: American Academy of Orthopaedic Surgeons Foot and Ankle Outcome Questionnaire Core Score
AAOS-SCS: American Academy of Orthopaedic Surgeons Foot and Ankle Outcome Questionnaire Shoe Comfort Scale
ADL: Activities of Daily Living
A-FORM: Ankle Fracture Outcome of Rehabilitation Measure
COSMIN: COnsensus-based Standards for the selection of health Measurement INstruments
EQ-5D: EuroQol EQ-5D Score
FAAM: Foot and Ankle Ability Measure
FAAM-ADL: Foot and Ankle Ability Measure Activities of Daily Living subscale
FAAM-S: Foot and Ankle Ability Measure Sports Subscale
FAOS: KOOS Foot and Ankle Outcome Score
GRADE: Grading of Recommendations Assessment, Development and Evaluation
LEFS: Lower Extremity Functional Scale
MOXFQ: Manchester Oxford Foot Questionnaire
N/A: Not Applicable
N/S: Not Specified
OMAS: Olerud Molander Ankle Score
PROMs: Patient Reported Outcome Measures
QoL: Quality of Life
SEFAS: Self Reported Foot and Ankle Score
SF-12: Short Form 12 Questionnaire
SF-12-MCS: Short Form 12 Mental Component Score
SF-12-PCS: Short Form 12 Physical Component Score
SF-36: Short Form 36 Questionnaire
SF-36-MCS: Short Form 36 Mental Component Score
SF-36-PCS: Short Form 36 Physical Component Score
