# Supplementary material for: A systematic review of the measurement properties of patient reported outcome measures used for adults with an ankle fracture
Source: J Patient Rep Outcomes. 2019 Dec 17;3:70. doi: 10.1186/s41687-019-0159-5 (PMC6917678; doi:10.1186/s41687-019-0159-5)
Supplement: Supplementary file 1 — Additional file 1. Search strategies. [file 41687_2019_159_MOESM1_ESM.docx]

Search Strategy MEDLINE

| Number | Term | Returns |
| --- | --- | --- |
| 1 | exp ANKE JOINT/ or exp ANKLE/ or exp ANKLE INJURIES/ or ankle.mp. or exp Fibula/ or exp TIBIA/ | 89752 |
| 2 | exp Ankle Fractures/ or exp Tibial Fractures/ or distal tibia fracture.mp. or distal fibula fracture.mp. | 15689 |
| 3 | exp Fractures, Bone/ or fracture*.mp. | 256133 |
| 4 | (Olerud Molander Ankle Score or OMAS or "Olerud and Molander Ankle Score" or MOXFQ or Manchester-Oxford Foot Questionnaire or M-OXFQ or AAOS or "AAOS Foot and Ankle Outcome*" or "AAOS Foot and Ankle Outcomes Questionnaire" or American Academy of Orthopaedic Surgeons).mp. [mp=title, abstract, original title, name of substance word, subject heading word, floating sub-heading word, keyword heading word, protocol supplementary concept word, rare disease supplementary concept word, unique identifier, synonyms] | 1079 |
| 5 | (Foot Function Index or FFI or “Foot and Ankle Measure” or FAAM or Karlsson Score or A-FORM or “Ankle Fracture Outcome of Rehabilitation Measure” or “Foot and Ankle Outcome Survey” or “KOOS Foot and Ankle Outcome Survey”).mp. [mp=title, abstract, original title, name of substance word, subject heading word, floating sub-heading word, keyword heading word, protocol supplementary concept word, rare disease supplementary concept word, unique identifier, synonyms] | 27204 |
| 6 | 1 and 3 | 16396 |
| 7 | 6 or 2 | 25910 |
| 8 | 4 or 5 | 28271 |
| 9 | 7 and 8 | 163 |
| 10 | (Validation Studies or Comparative Study).pt. | 1901079 |
| 11 | exp Psychometrics/ | 70442 |
| 12 | psychometr*.ti,ab. | 35491 |
| 13 | (clinimetr* or clinometr*).tw. | 820 |
| 14 | exp "Outcome Assessment (Health Care)"/ | 992383 |
| 15 | outcome assessment.ti,ab. | 2979 |
| 16 | outcome measure*.tw. | 184681 |
| 17 | exp Observer Variation/ | 40491 |
| 18 | observer variation.ti,ab. | 958 |
| 19 | exp Health Status Indicators/ | 283741 |
| 20 | exp "Reproducibility of Results"/ | 375090 |
| 21 | reproducib*.ti,ab. | 129497 |
| 22 | exp Discriminant Analysis/ | 9959 |
| 23 | (reliab* or unreliab* or valid* or coefficient or homogeneity or homogeneous or internal consistency).mp. [mp=title, abstract, original title, name of substance word, subject heading word, floating sub-heading word, keyword heading word, protocol supplementary concept word, rare disease supplementary concept word, unique identifier, synonyms] | 1082636 |
| 24 | (cronbach* and (alpha or alphas)).ti,ab. | 16389 |
| 25 | (item and (correlation* or selection* or reduction*)).ti,ab. | 16621 |
| 26 | (agreement or precision or imprecision or precise values or test-retest).mp. [mp=title, abstract, original title, name of substance word, subject heading word, floating sub-heading word, keyword heading word, protocol supplementary concept word, rare disease supplementary concept word, unique identifier, synonyms] | 312116 |
| 27 | (test and retest).ti,ab. | 21101 |
| 28 | (reliab* and (test or retest)).ti,ab. | 70425 |
| 29 | (stability or interrater or inter-rater or intrarater or intra-rater or intertester or inter-tester or intratester or intra-tester or interobserver or inter-observer or intraobserver or intraobserver or intertechnician or inter-technician or intratechnician or intra-technician or interexaminer or inter-examiner or intraexaminer or intra-examiner or interassay or inter-assay or intraassay or intra-assay or interindividual or inter-individual or intraindividual or intra-individual or interparticipant or inter-participant or intraparticipant or intra-participant or kappa or kappas or repeatab*).mp. [mp=title, abstract, original title, name of substance word, subject heading word, floating sub-heading word, keyword heading word, protocol supplementary concept word, rare disease supplementary concept word, unique identifier, synonyms] | 546799 |
| 30 | ((replicab* or repeated) and (measure or measures or findings or result or results or test or tests)).ti,ab. | 160445 |
| 31 | (generaliza* or generalisa* or concordance).ti,ab. | 66204 |
| 32 | (intraclass and correlation*).ti,ab. | 18505 |
| 33 | (discriminative or known group or factor analysis or factor analyses or dimension* or subscale*).ti,ab. | 449050 |
| 34 | (multitrait and scaling and (analysis or analyses)).ti,ab. | 126 |
| 35 | \| (item discriminant or interscale correlation* or error or errors or individual variability).ti,ab. \|  \| \| --- \| --- \| \|  \|  \| | 224152 |
| 36 | (variability and (analysis or values)).ti,ab. | 78802 |
| 37 | (uncertainty and(measurement or measuring)).ti,ab. | 4578 |
| 38 | (standard error of measurement or sensitive* or responsive*).mp. [mp=title, abstract, original title, name of substance word, subject heading word, floating sub-heading word, keyword heading word, protocol supplementary concept word, rare disease supplementary concept word, unique identifier, synonyms] | 1586719 |
| 39 | ((minimal or minimally or clinical or clinically) and (important or significant or detectable) and (change or difference)).ti,ab. | 182851 |
| 40 | (small* and (real or detectable) and (change or difference)).ti,ab. | 5693 |
| 41 | (meaningful change or ceiling effect or floor effect or Item Response model or IRT or Rasch or Differential item functioning or DIF or computer adaptive testing or item bank or cross-cultural equivalence).mp. [mp=title, abstract, original title, name of substance word, subject heading word, floating sub-heading word, keyword heading word, protocol supplementary concept word, rare disease supplementary concept word, unique identifier, synonyms] | 10118 |
| 42 | 8 or 9 or 10 or 11 or 12 or 13 or 14 or 15 or 1or 17 or 18 or 19 or 20 or 21 or 22 or 23 or 24 or 25 or 26 or 27 or 28 or 29 or 30 or 31 or 32 or 33 or 34 or 35 or 36 or 37 or 38 or 39 | 6236620 |
| 43 | 9 and 42 | 140 |

Search Strategy EMBASE

| Number | Term | Returns |
| --- | --- | --- |
| 1 | exp ankle/ or exp ankle injury/ or exp tibia/ or exp distal tibia/ or exp fibula/ or exp distal fibula/ | 97303 |
| 2 | ankle fracture.mp. or exp ankle fracture/ or exp distal tibial fracture or exp distal fibula fracture/ | 6738 |
| 3 | (distal tibia fracture or distal fibula fracture).mp. [mp=title, abstract, heading word, drug trade name, original title, device manufacturer, drug manufacturer, device trade name, keyword, floating subheading word, candidate term word] | 896 |
| 4 | fracture*.mp. | 402924 |
| 5 | 1 and 4 | 21885 |
| 6 | 2 or 3 or 5 | 22590 |
| 7 | (OMAS or Olerud Molander Ankle Score or “Olerud and Molander ankle Score” or Manchester-Oxford Foot Questionnaire or M-OXFQ or MOXFQ or AAOS or “AAOS Foot and Ankle Outcome Questionnaire” or “ American Academy of Orthopaedic Surgeons Foot and Ankle*”).mp. [mp=title, abstract, heading word, drug trade name, original title, device manufacturer, drug manufacturer, device trade name, keyword, floating subheading word, candidate term word] | 1214 |
| 8 | (Foot Function Index or FFI or “Foot and Ankle Measure” or FAAM or Karlsson Score or A-FORM or “Ankle Fracture Outcome of Rehabilitation Measure” or “Foot and Ankle Outcome Survey” or KOOS Foot and Ankle Outcome Survey”).mp. [mp=title, abstract, heading word, drug trade name, original title, device manufacturer, drug manufacturer, device trade name, keyword, floating subheading word, candidate term word] | 42789 |
| 9 | 7 or 8 | 43981 |
| 10 | 6 and 9 | 187 |
| 11 | exp intermethod comparison/ | 246249 |
| 12 | exp data collection method/ | 965974 |
| 13 | exp validation study/ | 75943 |
| 14 | exp feasibility study/ | 107128 |
| 15 | exp psychometry/ | 88700 |
| 16 | exp reproducibility/ | 203995 |
| 17 | (reproducib* or audit or psychometr* or clinimetr* or cliniometr*).abti. | 313777 |
| 18 | exp observer variation/ | 19458 |
| 19 | observer variation.ab,ti. | 1520 |
| 20 | exp discriminant analysis/ | 17496 |
| 21 | exp validity/ | 89705 |
| 22 | (reliab* or valid*or coefficient or internal consistency).ab,ti. | 1554337 |
| 23 | ((Cronbach* and (alpha or alphas)) or item correlations or item selection or item selections or item reduction or item reductions or agreement or precision or imprecision or precise values or test-retest or (test and retest)).ab,ti. | 488456 |
| 24 | ((reliab* and (test or retest)) or stability or interrater or inter-rater or intrarater or intra-rater or intertester or inter-tester or intratester or intra-tester or interobserver or inter-observer or intraobserver or intra-observer or intertechnician or inter-technician or intratechnician or intra-technician or interexaminer or inter-examiner or interassay or inter-assay or intraassay or intra-assay or interindividual or inter-individual or intra-individual or intraindividual or inter-participant or interparticipant or intraparticipant or intra-participant).ab,ti. | 631619 |
| 25 | (kappa or kappas or coefficient of variation or repeatab* or ((replica* or repeated) and (measure or measures or findings or result or results or test or tests))).ab,ti. | 431174 |
| 26 | (generaliza* or generalisa* or concordance or (intraclass and correlation*)).ab,ti. | 135185 |
| 27 | (discriminative or known group or factor analysis or facture analyses or facture structure or facture structures or dimensionality or subscale* or multitrait scaling analysis or multitrait scaling analyses).ab,ti. | 133287 |
| 28 | (item discriminant or interscale correlation or interscale correlations or ((error or errors) and (measure* or correlat* or evaluat* or accuracy or accurate or precision or mean)) or individual variability or interval variability or rate variability or variability analysis or (uncertainty and (measurement or measuring))).ab,ti. | 258810 |
| 29 | (standard error or measurement or sensitive* or responsive* or (limit and detection) or minimal detectable concentration or interpretab* or (small* and (real or detectable) and (change or difference)) or meaningful change or minimal important change or minimal important difference or minimally important change or minimally important difference or minimal detectable change or minimal detectable difference or minimally detectable change or minimally detectable difference or minimal real change or minimal real difference or minimally real change or minimally real difference or ceiling effect or floor effect or item response model or irt or rasch or differential item functioning or dif or computer adaptive testing or item bank or cross-cultural equivalence).ab,ti. | 1979250 |
| 30 | 11 or 12 or 13 or 14 or 15 or 16 or 17 or 18 or 19 or 20 or 21 or 22 or 23 or 24 or 25 or 26 or 27 or 28 or 29 | 5727752 |
| 31 | 10 and 30 | 89 |

Search Strategy - CINAHL

| **Number** | **Term** | **Returns** |
| --- | --- | --- |
| 1 | (MH “Ankle”) OR (MH “Ankle Injuries+”) OR (MH “Ankle Joint”) | 13517 |
| 2 | (MH “Tibia”) | 6803 |
| 3 | (MH “Fibula”) | 1319 |
| 4 | (MH “Fractures+”) | 48955 |
| 5 | “fracture*” | 71366 |
| 6 | 1 OR 2 OR 3 | 20515 |
| 7 | 4 OR 5 | 71617 |
| 8 | 6 AND 7 | 4019 |
| 9 | (MH “Tibial Fractures+”) OR (MH “Ankle Fractures”) OR (MH “Fibula Fractures”) | 4503 |
| 10 | 8 OR 9 | 6469 |
| 11 | “(Olerud Molander Ankle Score or OMAS or “Olerud and Molander Ankle Score”) OR (MOXFQ or Manchester-Oxford Foot Questionnaire or M-OXFQ) OR (“AAOS” or “AAOS Foot and Ankle Outcome Questionnaire” or “American Academy of Orthopaedic Surgeons Foot and Ankle*”)” | 435 |
| 12 | “Foot Function Index or FFI or “Foot and Ankle Measure” or FAAM or Karlsson Score or A-FORM or “Ankle Fracture Outcome of Rehabilitation Measure” or “Foot and ankle Outcome Survey” or “KOOS Foot and Ankle Outcome Survey”” | 0 |
| 13 | (MH “Psychometrics”) or ( TI psychometr* or AB psychometr* ) or ( TI clinimetr* or AB  clinimetr* ) or ( TI clinometr* OR AB clinometr* ) or (MH “Outcome Assessment”) or ( TI  outcome assessment or AB outcome assessment ) or ( TI outcome measure* or AB outcome  measure* ) or (MH “Health Status Indicators”) or (MH “Reproducibility of Results”) or (MH  “Discriminant Analysis”) or ( ( TI reproducib* or AB reproducib* ) or ( TI reliab* or AB reliab* )  or ( TI unreliab* or AB unreliab* ) ) or ( ( TI valid* or AB valid* ) or ( TI coefficient or AB  coefficient ) or ( TI homogeneity or AB homogeneity ) ) or ( TI homogeneous or AB  homogeneous ) or ( TI “coefficient of variation” or AB “coefficient of variation” ) or ( TI “internal  consistency” or AB “internal consistency” ) or (MH “Internal Consistency+”) or (MH  “Reliability+”) or (MH “Measurement Error+”) or (MH “Content Validity+”) or “hypothesis  testing” or “structural validity” or “cross-cultural validity” or (MH “Criterion-Related Validity+”) or  “responsiveness” or “interpretability” or ( TI reliab* or AB reliab* ) and ( (TI test or AB test) OR  (TI retest or AB retest) ) or ( TI stability or AB stability ) or ( TI interrater or AB interrater ) or ( TI  inter-rater or AB inter-rater ) or ( TI intrarater or AB intrarater ) or ( TI intra-rater or AB intrarater  ) or ( TI intertester or AB intertester) or (TI inter-tester or AB inter-tester) or ( TI intratester  or AB intratester) or ( TI intra-tester or AB intra-tester) or ( TI interobserver or AB  interobserver) or (TI inter-observer or AB inter-observer ) or ( TI intraobserver or AB  intraobserver) or ( TI intra-observer or AB intra-observer) or ( TI intertechnician or AB  intertechnician) or (TI inter-technician or AB inter-technician) or ( TI intratechnician or AB  intratechnician ) or ( TI intra-technician or AB intra-technician ) or ( TI interexaminer or AB  interexaminer ) or (TI inter-examiner or AB inter-examiner) or (TI intraexaminer or AB  intraexaminer ) OR (TI intra-examiner or AB intra-examiner ) or (TI intra-examiner or AB intraexaminer  ) or (TI interassay or AB interassay ) or ( TI inter-assay or AB inter-assay ) or ( TI  intraassay or AB intraassay) or ( TI intra-assay or AB intra-assay ) or (TI interindividual or AB  interindividual) or (TI inter-individual or AB inter-individual) OR (TI intraindividual or AB  intraindividual) or (TI intra-individual or AB intra-individual) or (TI interparticipant or AB  interparticipant) or (TI inter-participant or AB inter-participant ) or (TI intraparticipant or AB  intraparticipant) or (TI intra-participant or AB intra-participant ) or (TI kappa or AB kappa) or (TI  kappa’s or AB kappa’s ) or (TI kappas or AB kappas) or (TI repeatab* or AB repeatab*) or ( TI  responsive* or AB responsive* ) or ( TI interpretab* or AB interpretab* ) | 583534 |
| 14 | 11 AND 13 | 140 |
